# Urinary CXCL1: A Novel Predictor of IgA Nephropathy Progression

**DOI:** 10.1371/journal.pone.0119033

**Published:** 2015-03-27

**Authors:** Yanfeng Zhao, Li Zhu, Tong Zhou, Qingxian Zhang, Sufang Shi, Lijun Liu, Jicheng Lv, Hong Zhang

**Affiliations:** 1 Renal Division, Department of Medicine, Peking University First Hospital, Beijing, China; 2 Peking University Institute of Nephrology, Beijing, China; 3 Key Laboratory of Renal Disease, Ministry of Health of China, Beijing, China; 4 Key Laboratory of Chronic Kidney Disease Prevention and Treatment, Peking University, Ministry of Education, Beijing, China; 5 Renal Division, Department of Medicine, The First People’s Hospital of Aksu District, Xinjiang, China; Radboud University Medical Center, NETHERLANDS

## Abstract

**Background:**

IgA nephropathy (IgAN) is the most common form of primary glomerulonephritis worldwide. In recent years, consistent efforts have been made to develop new non-invasive biomarkers for IgAN progression. In our previous in vitro study we found mesangial derived CXCL1 as a contributor for kidney injury, and observed higher urinary CXCL1 levels in patients with IgAN. It implied that the urinary CXCL1 might be a potential biomarker.

**Methods:**

In the present study, we enrolled 425 IgAN patients with follow-up data and detected their urinary CXCL1 levels at the time of renal biopsy, to explore the predictive value of urinary CXCL1 in IgAN progression. Urinary CXCL1 levels were measured using enzyme-linked immunosorbent assay.

**Results:**

Urinary CXCL1 levels were associated with presently well established predictors of IgAN progression, including SBP (r = 0.138, p = 0.004), DBP (r = 0.114, p = 0.019), proteinuria (r = 0.155, p = 0.001), eGFR (r = -0.259, p<0.001) and tubular atrophy and interstitial fibrosis (r = 0.181, p<0.001). After adjusted for them, higher levels of urinary CXCL1 were independently associated with a greater risk of deterioration in renal function (HR, per s.d. increment of natural log–transformed CXCL1: 1.748; 95% CI: 1.222–2.499, P = 0.002). Furthermore, time-dependent receiver operating characteristic (ROC) curve showed that urinary CXCL1, when combined with proteinuria and eGFR, could enhance the prognostic value of these traditional predictors for IgAN progression.

**Conclusions:**

The results in our present study suggested urinary CXCL1 as a new non-invasive predictor of IgAN progression.

## Introduction

Immunoglobulin A nephropathy (IgAN) is the most common form of primary glomerulonephritis (GN) worldwide [1], characterized by mesangial IgA deposition. Patients with IgAN may appear with a variety of clinical manifestations and histological lesions. Moreover, the prognosis of IgAN is highly variable, ranging from sustained asymptomatic hematuria to rapid progression to end stage renal disease [2, 3]. Therefore, identification of patients at high risk of progression would be valuable for patient’s management in clinical practice. At present, clinical prognostic features indicating the well-known predictors for progression in IgAN include sustained hypertension, impaired renal function and persistent proteinuria. Histologically, renal biopsy is a vital means for IgAN diagnosis and outcome prediction. However, renal biopsy still has some limitations. First, it is an invasive examination, which precludes the repeated testing. Second, it reflects renal lesion only at the time point of biopsy, missed its dynamic changes as the disease progressed. Third, since only tens of glomeruli can be used for examination, it should be more cautious to use it to reflect the whole renal lesion, especially in case of focal other than diffused lesions. Thus, non-invasive bio-markers, which can dynamically reflect the whole renal lesion, are in urgent need.

In recent years, consistent efforts have been made to develop new non-invasive biomarker for acute and chronic kidney diseases. Approximately 70% of the urinary proteins are from the kidney and urinary tract, and therefore urine becomes a promising bio-sample for biomarker identification in patients with kidney disease. Recently, there are several studies devoted in urinary biomarker for IgAN, such as epidermal growth factor (EGF) [4], monocyte chemoattractant protein-1 (MCP-1) [5], EGF/MCP-1 ratio [6], complement (C3a and C5a) [7], Laminin G-like 3 and free κ light chains [8], and interleukin (IL)-1β,-2,-17,-6,-10 and Interferon (INF)-γ [5]. This kind of studies greatly enriched our knowledge about IgAN progression, although most of them need further validation because of limited sample size or their retrospective studies.

We previously reported that IgA1 complexes from IgAN patients could induce the up-regulation of chemokine (C-X-C motif) ligand 1 (CXCL1) in mesangial cells. And further in vitro experiments proved the podocyte injury effect induced by mesangial derived CXCL1. Moreover, we observed significantly higher urinary CXCL1 levels in patients with IgAN than those in healthy controls, which implied urinary CXCL1 as a potential bio-marker for IgAN [9]. In the present work, we enrolled a cohort of IgAN patients with regular follow-up and explored whether it could improve the predictive value for IgAN progression when adding urinary CXCL1 onto presently used markers, in order to evaluate the predictive value of urinary CXCL1 as a non-invasive biomarker in IgAN progression.

## Materials and Methods

### Study population

The present study recruited 425 IgAN patients with regular follow-up in Peking University First Hospital. The IgAN diagnosis was based upon the presence of dominant IgA demonstration in mesangial area by immunofluorescence, and confirmed by light microscopy and electronic microscopy, as well as the lack of clinical or serological evidence of other inflammatory conditions, such as systemic lupus erythematosus, vasculitis, or Henoch–Schoenlein purpura. During follow-up, patients received the same therapy regimen that be treated according to the Kidney Disease: Improving Global Outcomes (KDIGO) Guideline. As the proteinuria>1g/d, ACE inhibitors or ARBs were provided and dosage-adjusted according to the changes of blood pressure that optimal control target of < 130/80mmHg. Proteinuria is higher than 1g for three to six months without relief, and estimated glomerular filtration rate (eGFR)>50 ml/min/1.73m^2^, steroids are recommended. Steroids, in combination of other immunosuppressive agents, like cyclophosphamide, mycophenolate mofetil or FK506, were appropriate for crescentic IgAN with rapid progression [10, 11].

For enrolled patients, clinical manifestations, including age, gender, blood pressure, serum creatinine and 24h urine protein excretion were collected from medical records. The eGFR was calculated using the Chronic Kidney Disease Epidemiology Collaboration equation [12]. All renal biopsy specimens were reviewed and graded by an independent pathologist who was blinded to patients’ data and outcomes. The Oxford classification of the patients were analyzed, which were defined of four pathology features: mesangial hypercellularity score (M; M0≤0.5, M1>0.5), the presence of endocapillary proliferation (E; E0: absent, E1: present), segmental glomerulosclerosis/adhesion (S; S0: absent, S1: present), and severity of tubular atrophy/interstitial fibrosis (T; T0: 25%, T1: 26–50%, T2>50%) [13].

In the present study, we also enrolled 40 patients with minimal change disease (MCD), 40 patients with membranous nephropathy (MN), 40 patients with primary focal segmental glomerulosclerosis (FSGS), and 40 patients with lupus nephritis (LN) as disease controls. Moreover, 74 healthy subjects whose urinary CXCL1 were detected in our previous study were enrolled as healthy controls [9].

The study protocol was reviewed and approved by the Ethics Committee of Peking University and written informed consent was obtained from all participants.

### Detection of urinary CXCL1 by ELISA

Early morning urine samples on the day of renal biopsy from recruited 425 patients, as well as on the day of follow-up visits of 6 patients, were centrifuged immediately at 1800rpm and 4°C for 5 minutes. Then supernatants were stored at -80°C until the time of assays.

Urinary CXCL1 were quantified by a standard sandwich ELISA assays using the DuoSet human CXCL1 ELISA kits (R&D Systems, Minneapolis, MN, USA), according to the manufacturer’s protocol. Urinary CXCL1 were calibrated against urine creatinine before the levels were compared.

### The correlation analysis of urinary CXCL1 levels and patients outcomes

At first, the correlation of urinary CXCL1 levels with clinical and pathological indicators at the time of renal biopsy were explored. And then, survival analysis was applied to evaluate whether urinary CXCL1 levels at biopsy could predict IgAN outcome. A composite end point, defined as 50% eGFR decline, ESRD or death, whichever occurred first, was used in the present study. ESRD was defined as eGFR < 15 ml/min per 1.73 m^2^ or need for renal replacement therapy (such as hemodialysis, peritoneal dialysis or renal transplantation), for the purpose of this study.

### Statistical analyses

Statistical analyses were performed by SPSS software (version 16.0; SPSS, Chicago, IL, USA). Normally distributed quantitative variables were expressed as mean ± standard deviation. For non-normally distributed variables, we used median and interquartile range (IQR). Categorical data were summarized as absolute frequencies and percentages. For Continuous variables, independent-samples *t* test was used if the data was in normal distribution, and if not, Mann-Whitney or Kruskal-Wallis test was performed. Categorical variables were compared using χ^2^ test. Spearman’s correlation was applied for analyzing correlation. Factors correlated with urinary CXCL1 were further explored using multivariable linear regression analysis. We derived cumulative kidney survival curves using the Kaplan Meier method, and analyzed differences between curves by log-rank test.

For the analysis of Cox proportional hazards models and time-dependent receiver operating characteristic (ROC) curve, survival package and suvivalROC package in R (Version number: 3.0.2) were used, respectively. Cox proportional hazards models were used to analyze the association of urinary CXCL1 levels and the composite outcome. Results were presented as hazard ratio (HR) and 95% confidence interval (CI). Urinary CXCL1 were highly skewed to the left in our patients, therefore, natural log transformation was used. Urinary CXCL1 was analyzed as a continuous variable with HRs calculated per s.d. increment of natural log–transformed CXCL1. The relationship between CXCL1 and risk of end point was examined in unadjusted and multivariable-adjusted Cox models. Additionally, time-dependent receiver operating characteristic (ROC) curve analysis was conducted to evaluate the prognostic value of CXCL1 for the composite end. Areas under the curve were calculated for CXCL1, eGFR, proteinuria. A two-tailed P-value less than 0.05 was considered statistically significant.

## Results

### Demographic and clinical characteristics of patients with IgAN

We defined the time of renal biopsy as baseline and the characteristics of patients with IgAN (n = 425) were summarized in Table 1. Among them, 219 (51.5%) were males and 206 (48.5%) were females. The mean age at renal biopsy of patient was 34.56±12.42 years. All the patients were regularly followed-up, with a follow-up time of 47.46±25.88 months. At the time of biopsy, the level of median proteinuria was 1.66 g/24h (IQR 0.85–3.24 g/24h) and average eGFR was 86.24±29.77 ml/min/1.73m^2^. The mean systolic blood pressure (SBP) was 123±15 mmHg, while diastolic blood pressure (DBP) was 79±12 mmHg. Mesangial hypercellularity (M1), endocapillary hypercellularity (E1), and segmental glomerulosclerosis (S1) were found in 79.8%, 54.8%, and 67.6% of patients, respectively. Tubular atrophy and interstitial fibrosis, 0% to 25% (T0), 26% to 50% (T1), and >50% (T2), were found in 68.1%, 21.2%, and 10.7% of patients, respectively. And the median urinary CXCL1 level was 18.29 pg/mg (IQR: 10.17–33.47 pg/mg). During the follow-up period, 408 (96.0%) patients received ACE inhibitors or ARBs therapy, 179 (42.1%) received oral corticosteroids and 119(28.0%) received other immunosuppressive agents. In total, 45 patients reached the composite end point of 50% eGFR decline (n = 32), ESRD (n = 34; 21 of them had a 50% eGFR decline with ESRD), or death (n = 5; one of them had a 50% eGFR decline before death).

**Table 1:** Baseline clinical and laboratory data and levels of urinary CXCL1 in patients with IgAN.

Characteristics	Mean ± S.D. or Median (IQR)	CXCL1 Median Level(18.29 pg/mg)		P Value ^a^
		Lower(n = 212)	Upper(n = 213)	
**Baseline**				
Age (year)	34.56 ± 12.42	32.46 ± 10.79	36.65 ± 13.55	<0.001
Gender (male)	219 (51.5%)	121 (57.1%)	98 (46.0%)	0.022
Urinary CXCL1^b^ (pg/mg)	18.29 (10.17, 33.47)	10.17 (6.52, 13.87)	33.35 (24.41, 51.18)	<0.001
Initial proteinuria (g/day)	1.66 (0.85, 3.24)	1.29 (0.73, 2.87)	1.99 (0.99, 3.50)	0.002
<0.3 (%)	17/425 (4.0%)	10/212 (4.7%)	7/213 (3.3%)	0.149
0.3–0.99 (%)	112/425 (26.4%)	64/212 (30.2%)	48/213 (22.5%)	
1.0–2.99 (%)	179/425 (42.1%)	88/212 (41.5%)	91/213 (42.7%)	
≥3.0 (%)	117/425 (27.5%)	50/212 (23.6%)	67/213 (31.5%)	
eGFR (ml/ min per 1.73 m^2^)	86.24 ± 29.77	92.72 ± 26.51	79.79 ± 31.45	<0.001
CKD Stages 1, 2, 3, and 4^c^	213(50.1%), 123(28.9%), 72(16.9%), 17(4.0%)	130(61.3%), 54(25.5%), 23(10.8%), 5(2.4%)	83(39.0%), 69(32.4%), 49(23.0%), 12(5.6%)	<0.001
SBP (mmHg)	123 ± 15	120 ± 15	125 ± 15	0.001
DBP (mmHg)	79 ± 12	77 ± 11	80 ± 12	0.002
Oxford classification ^d^ (%)				
M1	335 (79.8%)	167 (79.9%)	168 (79.6%)	0.942
E1	230 (54.8%)	115 (55.0%)	115 (54.5%)	0.914
S1	284 (67.6%)	134 (64.1%)	150 (71.1%)	0.127
T1/T2	89 (21.2%) / 45 (10.7%)	39 (18.7%) / 13 (6.2%)	50 (23.7%) / 32 (15.2%)	0.002
**Follow-up**				
Follow-up interval (month)	47.46 ± 25.88	52.16 ± 28.50	42.79 ± 22.07	<0.001
Treated with ACE inhibitors or ARBs (%)	408 (96.0%)	204 (96.2%)	204 (95.8%)	0.812
Treated with immunosuppressive agents (%)	119 (28.0%)	50 (23.6%)	69 (32.4%)	0.043
Treated with prednisone (%)	179 (42.1%)	79 (37.3%)	100 (46.9%)	0.043

Abbreviations: IgAN, IgA nephropathy; CKD, chronic kidney disease; SBP, systolic blood pressure; DBP, diastolic blood pressure; eGFR, estimate glomerular filtration rate; IQR, interquartile range; ACE, angiotensin-converting enzyme; ARB, angiotensin II receptor blocker; Oxford classification: mesangial hypercellularity score (M1>0.5), the presence of endocapillary proliferation (E1: present), segmental glomerulosclerosis/adhesion (S1: present), and severity of tubular atrophy/interstitial fibrosis (T1: 26–50%, T2>50%).

^a^ P Value was used to indicated the difference between CXCL1 lower group and upper group. A two-tailed P-value less than 0.05 was considered statistically significant.

^b^ Urinary CXCL1 was calibrated against urine creatinine.

^c^ CKD stage 1, 2, 3, and 4 were divided by eGFR≥90, 60–89, 30–59, and 15–29, respectively, according to KDOQI.

^d^ Oxford classification was developed by Working Group of the International IgA Nephropathy Network and the Renal Pathology Society.

### Urinary CXCL1 levels in IgAN and non-IgA glomerulonephritis

**Fig 1:**
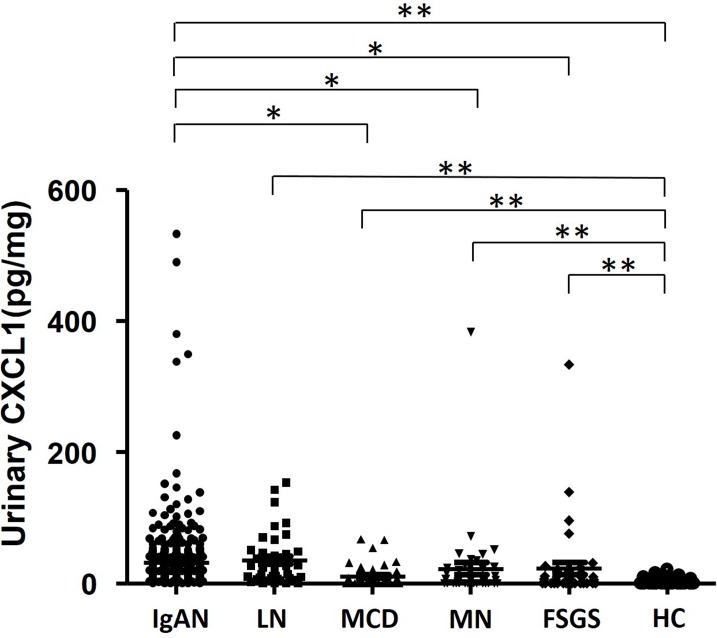
Urinary CXCL1 levels in IgAN and non-IgA glomerulonephritis. Urinary CXCL1 was calibrated against urine creatinine before the levels were compared. The urinary CXCL1 levels in patients with IgAN, MCD, MN, FSGS and LN were significantly higher than those in healthy controls (0, IQR 0–0, P<0.001 in all glomerulonephritis). Urinary CXCL1 levels were significantly higher in patients with IgAN (18.29 pg/mg, IQR 10.17–33.47 pg/mg) than in MCD (3.03 pg/mg, IQR 0–11.28 pg/mg, P<0.001), MN (5.89 pg/mg, IQR 0–23.54 pg/mg, P<0.001) and FSGS (6.10 pg/mg, IQR 0–17.53 pg/mg, P<0.001), while no significant difference was observed between patient with IgAN and LN (18.29 pg/mg, IQR 10.17–33.47 pg/mg vs. 23.12 pg/mg, IQR 7.41–48.17 pg/mg; P = 0.873). (*, significant difference between IgAN group and other GN groups; **, significant difference between healthy control group and disease groups).

Urinary CXCL1 levels were significantly higher in the patients of IgAN (18.29 pg/mg, IQR 10.17–33.47 pg/mg) than those in patients of MCD (3.03 pg/mg, IQR 0–11.28 pg/mg, P<0.001), MN (5.89 pg/mg, IQR 0–23.54 pg/mg, P<0.001) and FSGS (6.10 pg/mg, IQR 0–17.53 pg/mg, P<0.001), while not significantly higher than those in LN (23.12 pg/mg, IQR 7.41–48.17 pg/mg, P = 0.873) (Fig. 1). The urinary CXCL1 levels in patients of all these five diseases were significantly higher than those in healthy controls (0, IQR 0–0, P<0.001, all).

### Urinary CXCL1 levels correlated with severity of IgAN

A cross section correlation analysis between urinary CXCL1 levels and clinical and histological manifestations of IgAN patients at the time of renal biopsy was performed. The levels of CXCL1 in IgAN patients were positively correlated with proteinuria (r = 0.155, p = 0.001), SBP (r = 0.138, p = 0.004), DBP (r = 0.114, p = 0.019), tubular atrophy and interstitial fibrosis (r = 0.181, p<0.001), and negatively correlated with eGFR (r = -0.259, p<0.001). Patients with IgAN in severe CKD stages showed higher urinary CXCL1 levels (CKD 3–4: median 26.59 pg/mg, IQR 15.62–50.79 pg/mg vs CKD 1–2: median 16.67 pg/mg, IQR 9.17–29.77 pg/mg, p<0.001).

We divided the patients into two groups: according to the median level of urinary CXCL1 (18.29 pg/mg), and we found that compared with CXCL1 below median group, CXCL1 upper median group have higher initial proteinuria ((1.99, IQR 0.99–3.50) g/24h vs. (1.29, IQR 0.73–2.87) g/24h, P = 0.002, Table 1), lower eGFR (79.79±31.45 ml/min/1.73m^2^ vs. 92.72±26.51 ml/min/1.73m^2^, P<0.001), higher SBP (125±15 mmHg vs. 120±15 mmHg, P = 0.001),higher DBP (80±12 mmHg vs. 77±11 mmHg, P = 0.002),and more serious tubular atrophy and interstitial fibrosis (P = 0.002).

In order to identify the independent factors correlated with urinary CXCL1, we then applied multivariable linear regression model to our data. After adjusted for gender, age, eGFR, systolic blood pressure, diastolic blood pressure and tubular atrophy and interstitial fibrosis, proteinuria was independently associated with urinary CXCL1 levels (β = 0.128, P = 0.010, Table 2).

**Table 2:** Multivariable linear regression analyses for urinary CXCL1 level.

Parameters	Unstandardized β	Standardized β	P Value	95%CI
Age	0.476	0.114	0.042	0.017, 0.936
Gender	12.721	0.124	0.013	2.725, 22.717
Initial proteinuria	2.402	0.128	0.010	0.585, 4.220
eGFR	-0.123	-0.071	0.292	-0.353, 0.106
SBP	0.333	0.100	0.195	-0.172, 0.839
DBP	-0.402	-0.092	0.217	-1.041, 0.237
Tubular atrophy and interstitial fibrosis ^a^	7.635	0.101	0.082	-0.976, 16.246

Abbreviations: CI, confidence interval.

^a^ Tubular atrophy and interstitial fibrosis were classified by Oxford classification.

### Variation tendency of urinary CXCL1 during follow-up

**Fig 2:**
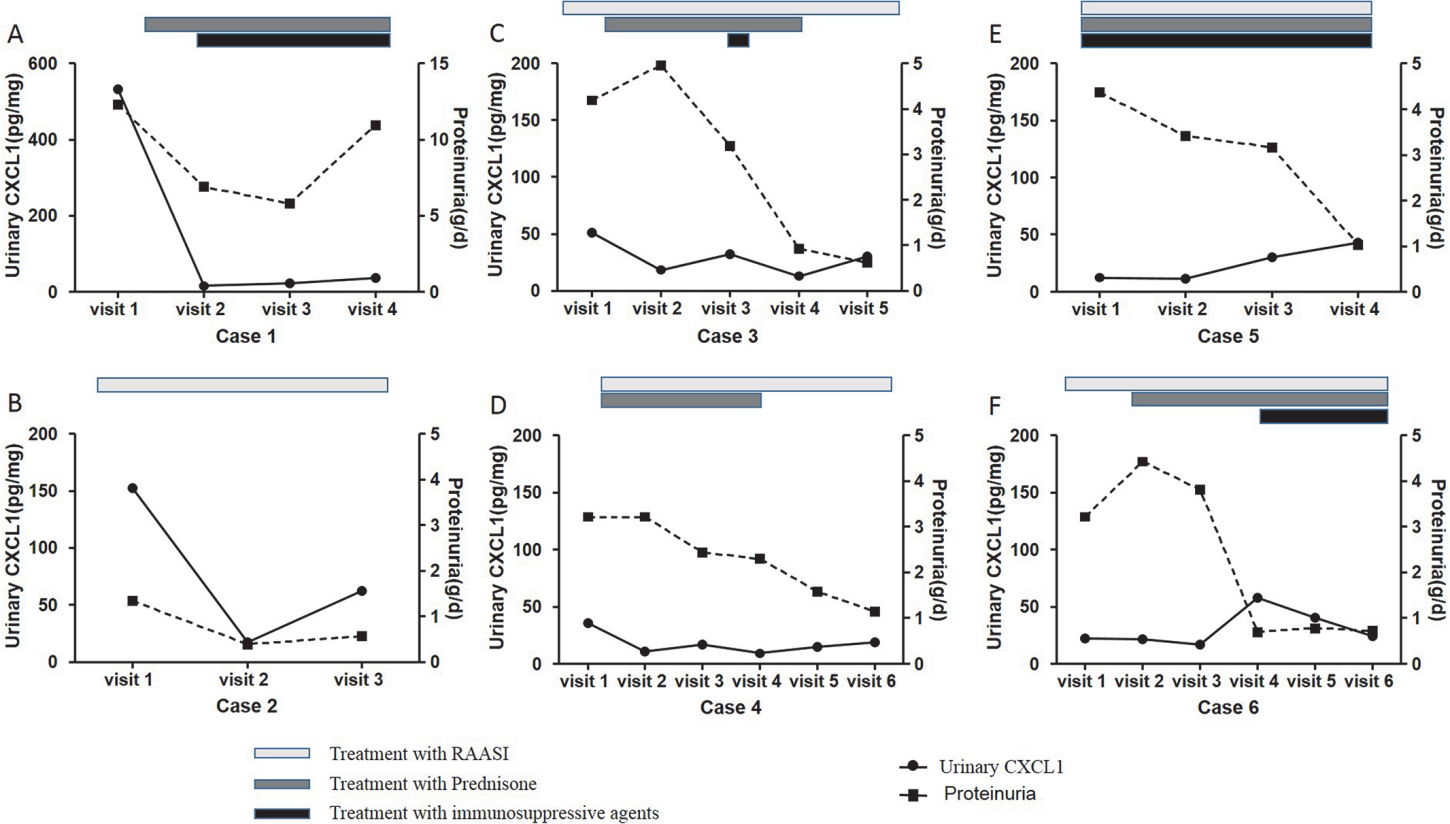
Correlation of urinary CXCL1 level with proteinuria during follow-up period. For patients with high baseline urinary CXCL1 levels (case 1 & case 2), the variation tendencies of urinary CXCL1 and proteinuria were almost the same (A & B). For patients with middle levels of baseline urinary CXCL1 (case 3 & case 4), although proteinuria relieved a lot, urinary CXCL1 levels just decreased a little (C & D). For patients with low levels of baseline urinary CXCL1 (case 5 & case 6), decreased urinary CXCL1 levels were not observed accompanied with proteinuria remission (E & F). Treatments are depicted as grey-level-coded bars, on behalf of the period of using time.

**Fig 3:**
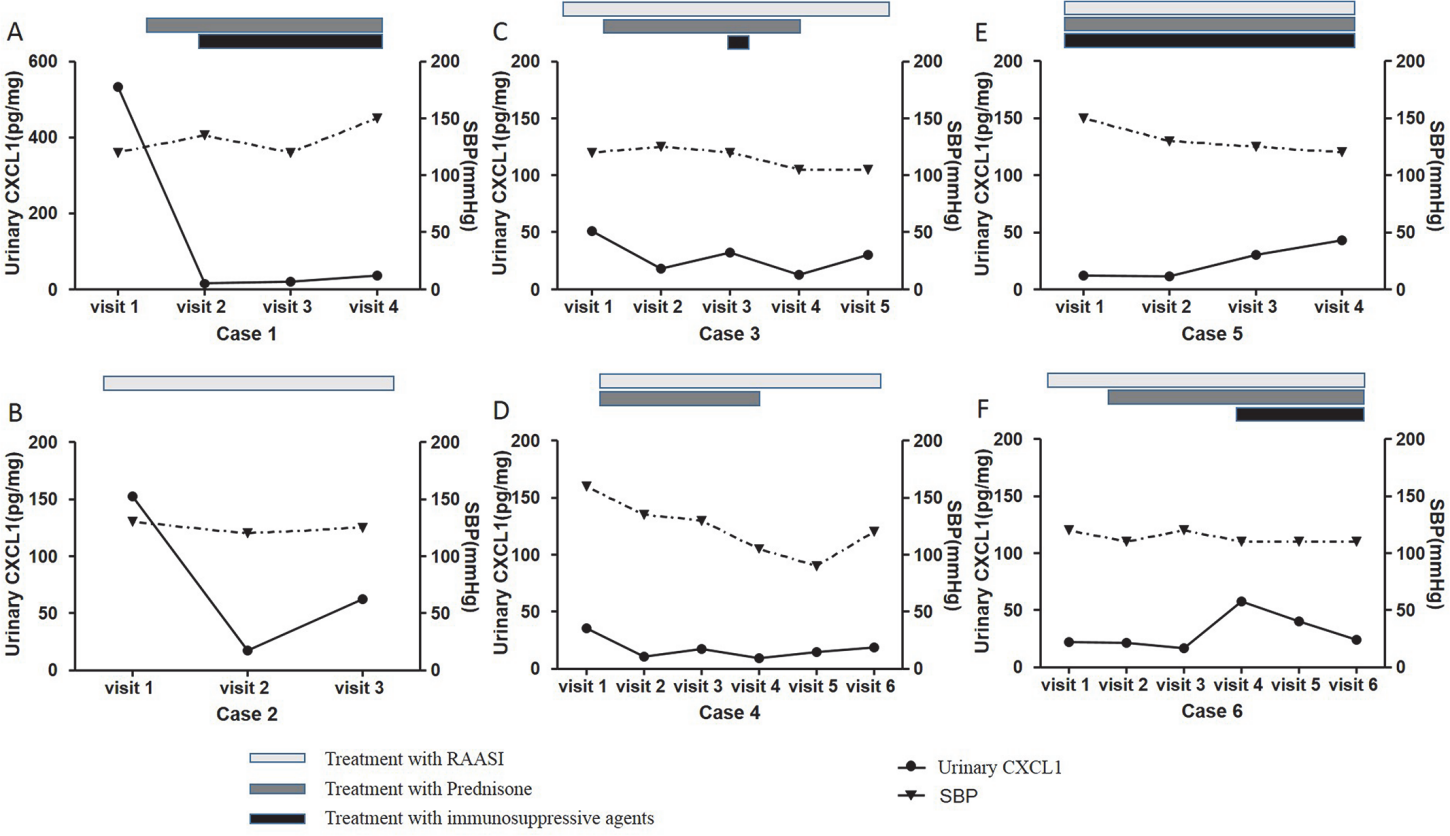
Correlation of urinary CXCL1 level with SBP during follow-up period. The variation tendency of urinary CXCL1 was not the same with that of SBP, not only in patients with high baseline urinary CXCL1 levels (case 1 & case 2, A & B), but also in patients with middle baseline urinary CXCL1 levels (case 3 & case 4, C & D) and patients with low baseline urinary CXCL1 levels (case 5 & case 6, E & F). Treatments are depicted as grey-level-coded bars, on behalf of the period of using time.

In order to evaluate the effect of treatment on urinary CXCL1, we collected sequential urine samples of 6 patients with IgAN. After treatment, all patients presented with varying degrees remission of proteinuria, while some had controlled blood pressure. However, we found that the variation tendency of urinary CXCL1 and proteinuria levels were not always the same. For patients with high baseline urinary CXCL1 levels (case 1 & case 2, Fig. 2A-B), the variation tendency of urinary CXCL1 was almost the same with that of proteinuria. For patients with middle levels of baseline urinary CXCL1 (case 3 & case 4, Fig. 2C-D), although proteinuria relieved a lot, urinary CXCL1 levels just decreased a little. For patients with low levels of baseline urinary CXCL1 (case 5 & case 6, Fig. 2E-F), decreased urinary CXCL1 levels were not observed accompanied with proteinuria remission. Regarding to blood pressure under treatment, the variation tendency of urinary CXCL1 was not the same with that of SBP (Fig. 3).

### Urinary CXCL1 levels correlated with progression of IgAN

In order to evaluate the prognostic effect of urinary CXCL1 for IgAN progression, we divided our patients into two groups according to the median level of urinary CXCL1 (18.29 pg/mg). Patients with CXCL1 levels less than 18.29 pg/mg were classified as group A, while others (CXCL1 levels above 18.29 pg/mg) were classified as group B. During follow-up, 11 patients (5.2%) in group A reached the composite outcome compared with 34 patients (16.0%) in group B (P<0.001; Table 3). Of these patients, 9 patients (4.2%) in group A had a 50% decline in eGFR compared with 23 patients (10.8%) in group B (P = 0.010). Moreover, end-stage renal disease occurred in 8 patients (3.8%) in group A and 26 patients (12.2%) in group B (P = 0.001). All of the 5 deaths were in group B (P = 0.061).

**Table 3:** Clinical outcomes according to urinary CXCL1 level.

			CXCX1 Below Median		CXCX1 Above Median		P Value
			(<18.29pg/mg)		(≥18.29pg/mg)		
Outcome	No.(%)	/100 patient-y	n(%)	/100 patient-y	n(%)	/100 patient-y	
50%decline in eGFR	32(7.5)	1.90	9(4.2)	0.98	23(10.8)	3.03	0.010
ESRD	34(8.0)	1.98	8(3.8)	0.86	26(12.2)	3.32	0.001
Death	5(1.18)	0.29	0(0)	0	5(2.35)	0.64	0.061
Composite	45(10.6)	2.68	11(5.2)	1.19	34(16.0)	4.48	<0.001

Note: Composite outcome included doubling of the baseline SCr concentration, ESRD, or death.

Abbreviations: ESRD, end-stage renal disease; SCr, serum creatinine.

**Fig 4:**
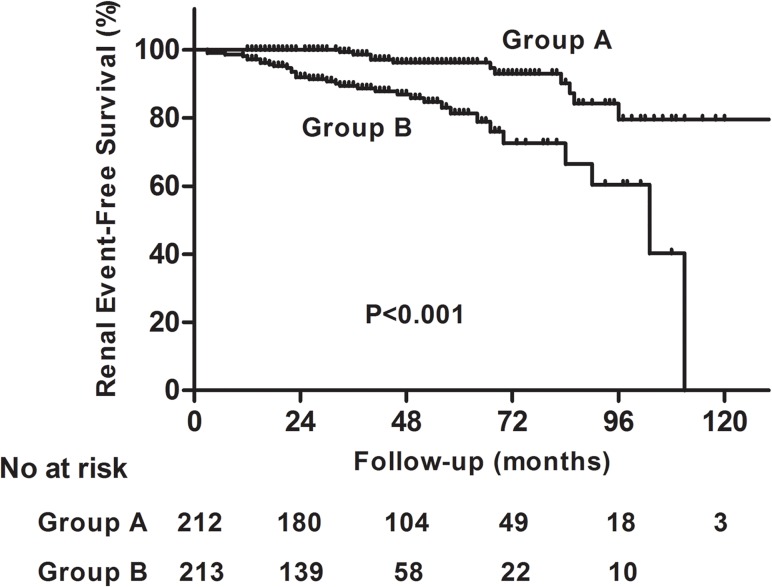
Kaplan-Meier renal survival curves of IgAN patients according to urinary CXCL1 levels. Patients with IgAN were divided into two groups, according to the median urinary CXCL1 level (18.29 pg/mg). Those with CXCL1 levels less than 18.29 pg/mg were classified as group A, while others (CXCL1 levels above 18.29 pg/mg) were classified as group B. IgAN patients in group B had significantly lower renal survival rate than those in group A (P < 0.001). The renal survival at first and fifth year for patients in group A were 100.0% and 96.3%, while for patients in group B were 97.2% and 81.3% (Log Rank test, p<0.001).

Kaplan-Meier survival analysis indicated that renal survival for the composite outcome was significantly lower in group B than that in group A (Log Rank test, p<0.001; Fig. 4). The renal survival rate for patients at first and fifth year were 100.0% and 96.3% for Group A, while 97.2% and 81.3% for Group B.

In Cox proportional hazards model, we at first tested baseline clinical and pathological variables for association with the composite progression outcome. In univariate analyses, lower baseline eGFR, higher proteinuria, higher systolic blood pressure, severer tubular atrophy and interstitial fibrosis, usage of steroids or other immunosuppressive agents were significantly associated with a poor renal outcome (Table 4). An increase in urinary CXCL1 levels was also significantly associated with reduced long term renal survival (HR, per s.d. increment of natural log–transformed CXCL1: 2.047; 95% CI: 1.487–2.816, P < 0.001). Moreover, after adjusting for well-established risk factors for IgAN (including baseline proteinuria, SBP, eGFR, tubular atrophy and interstitial fibrosis, and steroid or other immunosuppressive therapy), higher levels of urinary CXCL1 persistently showed significantly greater risk of kidney failure (HR, per s.d. increment of natural log–transformed CXCL1: 1.748; 95% CI: 1.222–2.499, P = 0.002, Table 5).

**Table 4:** Univariate analyses of clinical and pathological manifestations in Cox proportional hazards model.

	Univariate			Multivariate		
Parameter	HR	95% CI	P value	HR	95% CI	P value
Age	1.003	0.978–1.027	0.834	0.975	0.947–1.004	0.086
Gender	0.725	0.399–1.316	0.29	1.317	0.674–2.576	0.421
Natural log–transformed proteinuria	1.94	1.420–2.651	<0.001	1.151	0.761–1.739	0.506
eGFR	0.958	0.946–0.970	<0.001	0.972	0.957–0.986	<0.001
SBP	1.025	1.007–1.043	0.006	1.003	0.979–1.028	0.807
DBP	1.026	1.004–1.049	0.02	Not selected		
Pathologic measures (Oxiford classification)						
Mesangial hypercellularity score				Not selected		
M0 (≤0.5 of glomeruli)	1.000 (reference)	1.000 (reference)				
M1 (>0.5 of glomeruli)	0.968	0.493–1.902	0.924			
Endocapillary hypercellularity				Not selected		
E0 (absence)	1.000 (reference)	1.000 (reference)				
E1 (presence)	0.717	0.396–1.299	0.273			
Segmental glomerulosclerosis				Not selected		
S0 (absence)	1.000 (reference)	1.000 (reference)				
S1 (presence)	1.925	0.893–4.147	0.095			
Tubular atrophy and interstitial fibrosis						
T0 (≤25%)	1.000 (reference)	1.000 (reference)		1.000 (reference)	1.000 (reference)	
T1 (26%–50%)	3.066	1.415–6.645	0.005	1.491	0.643–3.459	0.352
T2 (>50%)	11.886	5.857–24.120	<0.001	3.793	1.501–9.588	0.005
RAAS inhibitors	1.056	0.144–7.739	0.958			
Prednisone and any other immunosuppressive agents	2.8	1.505–5.206	0.001	1.521	0.747–3.095	0.248
lnCXCL1	2.047	1.487–2.816	<0.001	1.748	1.222–2.499	0.002

Abbreviations: lnCXCL1, natural log–transformed CXCL1; HR, hazard ratio.

**Table 5:** Risks of composite end-point of natural log–transformed CXCL1.

	CXCL1, media (range)	Unadjusted	Hazard ratio (95% confidence interval) and P-value		
			Model 1^a^	Model 2^b^	Model 3^c^
Composite end point	18.29(0.00–533.11)	2.047 (1.487–2.816)	2.171(1.572–2.998)	1.737(1.221–2.471)	1.748(1.222–2.499)
Per 1 s.d lnCXCL1		<0.001	<0.001	0.002	0.002
CXCL1 subgroup					
A	10.17(0.00–18.29)	1(Reference)	1(Reference)	1(Reference)	1(Reference)
B	33.35(18.29–533.11)	4.624(2.309–9.260)	5.023(2.492–10.124)	2.879(1.407–5.892)	2.914(1.417–5.989)
		<0.001	<0.001	0.004	0.004

^a^ Model 1 adjusted for sexual and age. Sexual was analyzed as dichotomous data.

^b^ Model 2 adjusted for covariates in model 1 plus estimate glomerular filtration rate (eGFR), natural log–transformed proteinuria, systolic pressure and tubular atrophy and interstitial fibrosis. The latter variable was analyzed as categorical data.

^c^ Model 3 adjusted for covariates in model 2 plus steroid or other immunosuppressants use (yes or no). The latter variable was analyzed as dichotomous data.

Composite end point was defined as 50% decline of eGFR, end-stage renal disease, or death. Unadjusted model analyzed CXCL1 as continuous data.

We also conducted a separated analysis in which CXCL1 level was treated as a categorical variable. In accordance with the result above, the risk of reaching the composite outcome was significantly higher in group B patients than that in group A patients (HR, per s.d. increment of CXCL1: 2.914; 95% CI: 1.417–5.989; P = 0.004).

**Fig 5:**
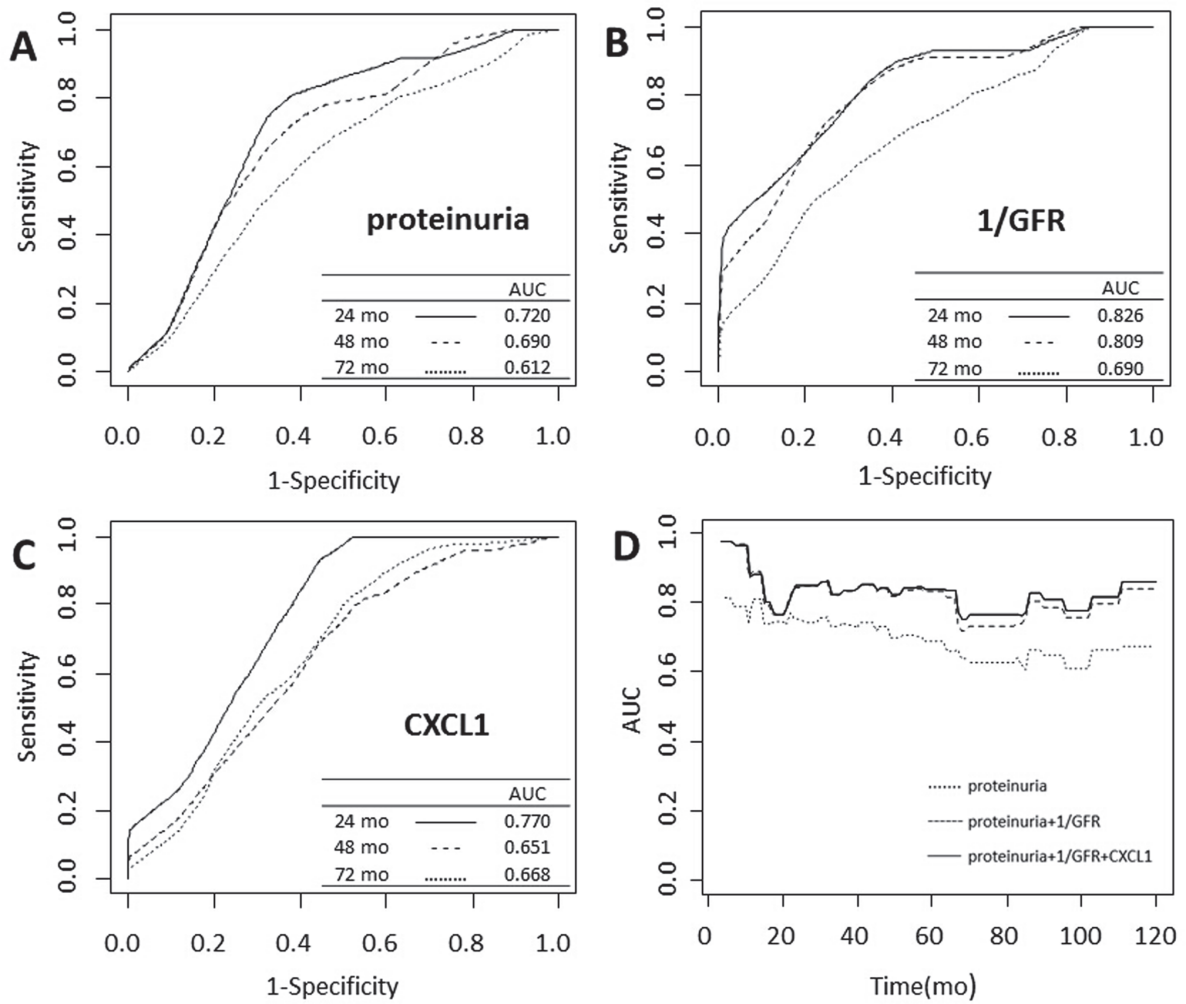
Time-dependent receiver operating characteristics curves with composite outcome as the status variable. Using composite outcome at 24 (solid line), 48 (dashed line), and 72 months (dotted line) as status variable, respectively, the areas under the ROC curve (AUC) for proteinuria (A), reciprocal of estimated glomerular filtration rate (1/eGFR) (B) and urinary CXCL1 (C) were comparable (proteinuria: 0.720 (24 mo), 0.690 (48 mo), 0.612 (72 mo); 1/eGFR: 0.826 (24 mo), 0.809 (48 mo), and 0.690 (72 mo); urinary CXCL1: 0.770 (24 mo), 0.651 (48 mo), 0.668 (72 mo)). When combined urinary CXCL1 level with proteinuria and 1/eGFR (solid line), the AUCs were higher than proteinuria alone (dotted line) or proteinuria plus 1/eGFR (dashed line) (D).

To evaluate the operating characteristics of urinary CXCL1 level as a prognostic value for IgAN progression, we conducted a time-dependent ROC analysis for urinary CXCL1 level in comparison with proteinuria and reciprocal of eGFR (1/eGFR) level (Fig. 5). The areas under the ROC curve (AUC) for CXCL1 at 24, 48, and 72 months were 0.770, 0.651, and 0.668, respectively, which were comparable to those for proteinuria and 1/eGFR. Furthermore, after combined urinary CXCL1 level, proteinuria and 1/eGFR together, the AUCs were higher than single clinical parameters (proteinuria or 1/eGFR) or in their combination (proteinuria plus 1/eGFR) (Fig. 5).

In order to further evaluate the additive effect of urinary CXCL1 on traditional predictive markers for IgAN progression, we carried out the survival analysis by using both base model (M0: including our previous well known markers for IgAN progression, including eGFR, proteinuria, blood pressure and pathological features) and a new model, in which urinary CXCL1 were added (M1 = M0+CXCL1). The results showed that with addition of urinary CXCL1, the overall performance of the predictive model for IgAN progression improved (R square: M1 Vs M0: 0.182 Vs 0.164, p = 0.002, Table 6).

**Table 6:** Comparison of two models of survival analysis.

Model	R-square	AIC	Models compared	P-value (LR*test)
M0: Age+Gender+eGFR+Proteinuria+SBP+T lesion ^a^	0.164	390.2341	—	—
M1: M0+CXCL1 ^b^	0.182	383.0455	M1 vs M0	0.002

^a^ Proteinuria, natural log–transformed Proteinuria.

^b^ CXCL1, natural log–transformed CXCL1.

## Discussion

CXCL1, also known as growth-regulated oncogene-alpha, is one of the CXC chemotactic factors [14–16]. It plays a key role in inflammation through its receptors, CXCR2, which is expressed on both inflammatory cells and non-inflammatory cells [17–20], including kidney podocytes [7]. Besides gastric, colon and skin cancers [21, 22], up-regulated CXCL1 has been reported in many different types of kidney diseases. In anti-MPO IgG-induced necrotizing crescentic glomerulonephritis, increased circulating CXCL1 protein levels were demonstrated [23]. Furthermore, elevated expression of CXCL1 was also reported in glomeruli of patients with focal segmental glomerulosclerosis obtained by laser capture microdissection [24]. However, reports regarding urinary CXCL1 and even its effect in IgAN were limited till today.

Our present study is the first clinical study evaluating the prognostic significance of urinary CXCL1 level on renal survival conducted in a cohort of IgAN patients with long-term follow-up. We found that urinary CXCL1 levels were significantly higher in patients with IgAN and LN than in other GN, including MCD, MN and FSGS. Since usually accompanied with other systemic symptoms, LN can be easily differentiated from IgAN. Therefore, although urinary CXCL1 cannot be used as a fully specific biomarker for IgAN among patients with primary nephritis, those with IgAN showed significantly higher urinary CXCL1 levels. Combined with other clinical parameters, urinary CXCL1 might be used as specific biomarker set for IgAN. Of course, the establishment of the specific biomarker set for IgAN needs further exploration and validation in the future studies.

In this study, we found that urinary CXCL1 levels were positively associated with 24h urinary protein excretion, SBP and tubular atrophy and interstitial fibrosis, and negatively associated with eGFR, all of which were well-established predictive markers for IgAN progression. Interestingly, when combined with baseline 1/eGFR and proteinuria, urinary CXCL1 could enhance the prognostic value over each individual clinical marker. The results were consistent with our previous finding in cross-sectional study, IgAN patients with severe clinical and pathological lesions presented with higher urinary CXCL1 levels [9]. In the next multivariable linear regression analysis, we found that 24h urinary protein excretion was independently associated with urinary CXCL1 level. Using in vitro mesangial cell culture model, we previously reported that mesangial derived CXCL1 could induce podocyte loss. Since increasing evidences proved podocyte damage as a leading cause for proteinuria [25], our present finding verified the important role for CXCL1 in podocyte injury in IgAN.

Following our identification that urinary CXCL1 level was independently associated with 24h urinary protein excretion, we further evaluated the dynamic changes of urinary CXCL1 during treatment, focused on those patients who presented with some degrees of proteinuria remission after treatment. Our observation in follow-up patients with sequential urine samples suggested that only for patients with high levels of baseline urinary CXCL1, the fluctuations of urinary CXCL1 levels were synchronized with those of proteinuria, implying that urinary CXCL1 above a certain level might participate in podocyte injury. Thereby, for those with high baseline levels, urinary CXCL1 could be served as a useful marker for not only IgAN progression, but also treatment efficacy, especially regarding proteinuria remission. However, our sequential urine samples were just available in a small proportion of patients. We presently have no chance to evaluate the correlation of proteinuria and urianry CXCL1 during follow-up in all patients, which awaiting further verification in future studies.

In order to evaluate the role of urinary CXCL1 in prediction of IgAN progression, COX regression model was used in our study. We demonstrated that urinary CXCL1 could serve as an independent prognostic factor for composite end point of IgAN, even after adjustment for baseline risk factors including proteinuria, eGFR, SBP and tubular atrophy and interstitial fibrosis. This result implied extra value of urinary CXCL1 over already established risk factors for the prediction of IgAN progression. Furthermore, we evaluated the predictive value of urinary CXCL1 in IgAN progression. With addition of urinary CXCL1 onto the traditional predictive markers for IgAN, the overall performance of the predictive model for IgAN progression improved significantly, which suggested that urinary CXCL1, although not fully specific, still could be served as a valuable non-invasive biomarker in predicting IgAN progression.

Regarding the underlying mechanism of CXCL1 in IgAN progression, we had little evidence, which was the limitation of our present study. CXCL1 was famous for its powerful neutrophil chemoattractant activity. However, in IgAN, the infiltrating inflammatory cells are mainly lymphocytes and monocytes, rather than neutrophils. Our previous study proved that up-regulated CXCL1, derived from mesangial cells under the challenge of pathogenic IgA1 complex, would increase podocyte death and reduce podocyte adhesion, and at last lead to podocyte loss [9]. In accordance with our finding, Lai KN et al. reported the decreased expression of nephrin in podocytes in IgAN [26]. In IgAN patients, Hara M et al. observed that cumulative excretion of urinary podocytes could reflect IgA nephropathy progression [27]. The podocyte was regarded as a central cell in glomerular disease. Damage and detachment of podocytes lead to foot processes retraction, resulting in proteinuria, even in glomerulosclerosis in later period. Immune injury induced podocyte loss would develop proteinuria, glomerulosclerosis, and progressive loss of kidney function [28]. Although it is very likely that CXCL1 contributed to IgAN progression through its induction of podocyte injury, direct evidence is still lacking today.

In conclusion, we showed that high urinary CXCL1 level is associated with severe clinical and histological findings, and poor renal prognosis in IgAN. When combined with well-established clinical risk factors, urinary CXCL1 may be a useful non-invasive biomarker for IgAN progression.
